# TBD

**DOI:** 10.1093/geroni/igab046.1948

**Published:** 2021-12-17

**Authors:** Tamara Baker

**Affiliations:** University of North Carolina, Chapel Hill School of Medicine, University of North Carolina at Chapel Hill (School of Medicine), North Carolina, United States

## Abstract

Chair: Lori Simon-Rusinowitz;Panelists: Representatives of GSA Sections - Phillip Rozario (SRPP), Stephen Helfand (BS), Tamara Baker (BSS), Cynthia Brown (HS), Judith Howe (AGHE), Darina Petrovsky (ESPO); Discussant: Brian Lindberg. This interactive session is an interdisciplinary look at policy issues in aging with the speakers representing views from their sections. This session, organized by the GSA Public Policy Panel, will provide both GSA section leadership and attendees an opportunity to have an open dialogue on important public policy issues of significance in the field of aging. Presentations will likely address the COVID-19 pandemic, how GSA, each section and its members, and the federal government responded during the past year; how the policy work has been influenced by the increased acknowledgement of institutional and societal racism; and member experiences with the influx of additional economic relief, research funding, and funding for aging supports and services programs.

